# What are the Experiences of and Interventions for Adult Survivors of Childhood Sexual Abuse in South Asia? A Systematic Review and Narrative Synthesis

**DOI:** 10.1177/15248380241231603

**Published:** 2024-02-22

**Authors:** Shivangi Talwar, Carlos Osorio, Rajesh Sagar, Rebecca Appleton, Jo Billings

**Affiliations:** 1 Division of Psychiatry, University College London, UK; 2 Department of Psychiatry, All India Institute of Medical Sciences, New Delhi, DL, India; 3 Talking Therapies Southwark, South London and Maudsley NHS Foundation Trust, UK

**Keywords:** adult survivors of childhood sexual abuse, systematic review, narrative synthesis, childhood sexual abuse, survivors of childhood sexual abuse, trauma, childhood trauma, adverse childhood experiences

## Abstract

Adult survivors of childhood sexual abuse (CSA) may experience emotional, social, and psychological difficulties, heightened due to the interpersonal nature of harm. Despite the demonstrated effectiveness of trauma-focused treatments in the West, a culturally specific understanding of the needs of and treatments for survivors in South Asia is still in its infancy. The study aimed to systematically review research findings on the mental health impacts of CSA on adult survivors and current treatment approaches and their efficacy and acceptability in South Asia. Seven databases (Scopus, Ovid, CINAHL, ProQuest, EThOS, Google Scholar, and Dogpile) and five peer-reviewed South Asian journals were searched from inception until March 30, 2023. Searches included participants who were adult survivors of CSA of South Asian origin residing in South Asia. Studies on their mental health, different treatments, and the efficacy and acceptability of these treatments were included. Quality assessment tools were used to appraise the quality of included studies. The results were synthesized narratively. A total of 3,362 records were retrieved, and 24 articles were included in the final review. Twenty studies reported mental health impacts of CSA on adult survivors, four studies reported current treatments offered, and two studies were on recovery. However, no study focused on the efficacy or acceptability of the treatments being delivered. Even though the needs of adult CSA survivors in South Asia have been partly identified, there is very little research into the treatments for them.

## Introduction

Childhood sexual abuse (CSA) can have specific and persistent consequences in adult life (Ehring et al., 2014) including difficulties in initiating and maintaining relationships as well as cognitive and emotional problems (Kia-Keating et al., 2010; McGregor et al., 2006). As it is often a repetitive and prolonged stressor during the developmental years, CSA has been classified as a form of complex trauma (Ford & Courtois, 2021). This, in turn, can put the adult survivor at risk of developing Complex Post-Traumatic Stress Disorder (CPTSD). A salient point is the interpersonal nature of harm caused by CSA, often leading to complex emotional responses to traumatic stressors, with ramifications in adult relationships (Coyle et al., 2014).

The International Classification of Diseases-11 criteria for Post-Traumatic Stress Disorder (PTSD) include three symptom clusters—reexperiencing the trauma, avoidance of traumatic reminders, and a persistent sense of current threat (World Health Organization, 2018). CPTSD, in addition to those, involves disturbances in self-organization: affective dysregulation, negative self-concept, and disturbances in relationships (World Health Organization, 2018).

First-line treatments for PTSD in adults in the United Kingdom (UK) are trauma-focused cognitive behavioral therapy and eye movement desensitization and reprocessing (EMDR) (NICE Guidelines, 2022). However, research into the psychological treatment of CPTSD is still in its initial stages. In Western countries, there is a growing evidence base demonstrating the effectiveness of trauma-focused treatments for adult survivors of CSA (Hegarty et al., 2022; Karatzias et al., 2022; Ringel, 2014). To date, non-Western contextual understanding and therapies for PTSD/CPTSD have been lacking. Simply transposing models from Western countries could omit the clients’ cultural patterns, backgrounds, and interpretation of a phenomenon (Bernal et al., 2009).

Cultural notions of CSA may inform adult survivors’ understanding of their experiences of CSA (Shafe & Hutchinson, 2014). Feelings of guilt, blame and shame, interpersonal difficulties, and mental health issues have been demonstrated in survivor research conducted with majorly White population in the United States of America (USA), the UK, Canada, Australia, and New Zealand. The need to understand survivors from “ethnic minorities” in these regions led to the identification of several cultural idiosyncrasies such as familial reputation among South Asian families (Sawrikar et al., 2017). Further, the USA, the UK, New Zealand, Canada, and Australia tend to have more individualistic cultures (Hofstede, 2001). On the other side of the spectrum, countries within Asia and Eastern Europe typically tend to be more collectivistic (Hofstede, 2001).

The reported prevalence of CSA is 1 in 4 girls and 1 in 13 boys in the USA (Centre for Disease Control and Prevention, 2022) and 1 in 20 children in the UK (National Health Services-NHS, 2023). Although a meta-analysis conducted on the prevalence of CSA globally found that most of the prevalence studies on CSA were from American and European continents (Pereda et al., 2009), Asia and Africa continue to demonstrate high prevalence rates of CSA (Selengia et al., 2020).

South Asia is the most populated region worldwide, with 2.5 children per woman. This region comprises Afghanistan, Pakistan, India, Sri Lanka, Bangladesh, Nepal, Bhutan, and the Maldives (World Bank, 2023). The South Asian region is home to an ancient civilization and has agricultural economies, and the religion- and caste-based structures impact their cultures (Anisuzzaman, 2013). Within South Asia, each country has their predominant religion, multiple languages, and ethnic groups (Anisuzzaman, 2013). They also have their own geopolitical and social problems, including natural disasters and economic and/or armed conflicts affecting daily lives within these regions (United Nations Children Fund—UNICEF, 2016). These factors could impact the development of mental health services in South Asian countries.

High rates of CSA have been reported across all South Asian countries and appear to be escalating. The reported figures have employed varied age criteria and have been conducted by national or international bodies or on a small scale by research teams. For example, Indian and Sri Lankan national bodies reported 31.2% and 735 child sexual offense cases respectively in 2015 (Rajkumar & Kirkpatrick, 2015; Rohanachandra et al., 2023). Nepalese statistics emerged from a study citing 33%–45% of school-going children as sexual assault victims (Basnet et al., 2020).

Pakistan and Bangladesh prevalence rates, reported by nongovernmental organizations, cited a 15% increase in CSA cases in 2018 over 2017, and 365 child victims of rape and sexual harassment in mid-2021, respectively (Mushtaque et al., 2022; Shoib et al., 2022). United Nations Children’s Fund (UNICEF) offices reported that 12.8% of children have experienced at least one sexual assault episode in their lives in Bhutan (UNICEF, 2016), and 1,200 cases of child abuse and assault were reported in 2019 in the Maldives (UNICEF Maldives Office, 2020). In the absence of a reliable source for CSA prevalence rates in Afghanistan, Children of Afghanistan-Humanium (2019) cited that years of upbringing in a war-torn region would put young people at constant risk of abuse. With repeatedly elevated occurrences of sexual abuse among children in South Asia, the number of adults with complex developmental histories will only increase. Nevertheless, there is a dearth of research into adult survivors’ experiences, treatment needs, and evidence-based treatments in South Asian countries.

We set out to systematically review and synthesize current published literature by answering two research questions: (a) What is the impact of CSA on the mental health of adult survivors in South Asia? and (b) Which treatment approaches have been offered to adult survivors of CSA in South Asia and what is the efficacy and acceptability of these approaches?

In this study, efficacy would determine if a treatment was likely to bring about desired outcomes for recipients under controlled conditions and acceptability would demonstrate if the survivors receiving the treatments find them appropriate or not (Sekhon et al., 2017).

## Methods

The review was prepared following the Preferred Reporting Items for Systematic Reviews and Meta-Analyses (PRISMA) guidelines (Page et al., 2021). Our review protocol was registered with the International Prospective Register of Systematic Reviews (PROSPERO) with Reg no. CRD42022306126.

### Search Strategy

We searched five databases namely Scopus, Ovid (Medline, Embase, and PsycInfo), CINAHL, ProQuest, and EThOS, from inception until March 30, 2023. Further, we hand-searched relevant journals including the *Indian Journal of Psychiatry*, *Sri Lanka Journal of Psychiatry*, *Journal of Pakistan Psychiatric Society*, *Journal of Psychiatrists’ Association of Nepal*, and *Bangladesh Journal of Psychiatry*. In addition, we searched our University’s online database, Google Scholar and Dogpile (which derives searches from engines such as Google and Yahoo). For results of searches conducted on journals, our University’s database, Google Scholar, and Dogpile, the limits were set to reviewing papers appearing in the first 10 pages of the search. This was done to ensure that we were including the most relevant records and that they were feasible for screening (Godin et al., 2015). However, considering the low number of results following these searches, the limit to including the first 10 pages applied only to the University’s database. The key search terms for both the review questions can be found in Table 1. We combined key search terms with MeSH terms, using Boolean operators where necessary.

**Table 1.** Key Search Terms.

Concept 1	Concept 2	Concept 3
Adult survivor* of child abuse	South Asia* or India* or Pakistan* or Afghan* or Sri Lanka* or Maldiv* or Bhutan* or Nepal* or Bangladesh*	treat*
Sexual abuse survivor*	Asia/or Bangladesh/or Bhutan/or India/or Nepal/or Pakistan/or Sri Lanka/	drug therap*
([molest* or sexual* abus*] adj6 childhood)		therap*.
([adult survivor* or adult victim*] adj4 sexual abuse).		pharmacotherap*.
child abuse, sexual/and adverse childhood experiences/		psychotherap*
		faith heal*
		spiritual therap*
		spiritual heal*
		spirit*
		mental heal*
		prayer heal*.
		restorative healing.
		“wounds and injuries”/or shock, traumatic/
		psychological trauma/or historical trauma/or sexual trauma/
		“behavioral disciplines and activities”/or exp behavior control/

*Selection criteria for studies and participants*: The inclusion criteria for studies involved (a) qualitative, quantitative and mixed methods research designs, (b) primary research studies, systematic reviews, treatment guidelines for PTSD/CPTSD drafted by competent authorities in South Asian countries, theses, and dissertations, and (c) published in English and South Asian languages. The included studies’ participants were (a) above 18 years of age, (b) had a history of sexual abuse in their childhood i.e., between 0 and 18 years of age, and (c) of South Asian ethnicity residing in South Asia.

Studies were excluded if they (a) were not in English or South Asian languages, (b) had participants from South Asian origin residing outside South Asia and, (c) had non-South Asian participants residing in South Asia. Studies were also excluded if participants were (a) below 18 years of age and (b) from South Asian origin but were receiving treatment from a non-South Asian country.

### Screening

Identified records were deduplicated and uploaded to Rayyan for screening (Ouzzani et al., 2016). We drafted a predetermined checklist for independent raters assessing the eligibility of a record at the title and abstract as well as at the full-text screening stage. Following the agreed protocol, ST and CO screened the same first 100 records independently and met to discuss any discrepancies. Then, they screened the remaining records independently, with CO reviewing 30% of the records. Systematic reviews and study protocols that were relevant to our review but not specific to our region of study were included for citation chaining only. All full texts were independently screened by both ST and CO. The authors of the records for which the full texts were not available were contacted to request the full paper. Any discrepancies in either of the screening stages were discussed with RA.

Data were extracted on MS Excel from included studies into a prepiloted data extraction form by ST. CO reviewed the data extracted for 10% of the texts included. Broadly, the form included information on the study site, aims and objectives of the study, sample characteristics, and key findings. Any missing information was noted as not reported. Citation chaining, both forward and backward, of all the included articles was conducted to ensure that any pertinent articles not found during the database searches were also retrieved.

### Quality Assessment

We assessed the quality of our included studies using Critical Appraisal Skills Program (CASP) Checklists for Qualitative studies and Case-Control studies (Critical Appraisal Skills Programme, 2022a, 2022b), the Appraisal Tool for Cross-Sectional Studies (Downes et al., 2016), and the Joanna Briggs Institute case report and case series (Moola et al., 2015) checklists.

The construction of assessments is directed by the race and ethnicity of the researchers, usually from Western contexts, and tested on a homogeneous group of participants (Rai et al., 2021). Hence, we decided to modify the assessment tools on two bases: (a) whether data collection included culturally validated tools and was conducted in participants’ preferred language and (b) whether researchers considered the sensitive nature of abuse in conducting research by employing risk assessments, safeguarding measures, and offering sources of support or any other ways of ensuring participants’ safety. The final items of the tools and quality ratings can be found in detail in Supplemental Appendices A–E.

We appreciate that the modified tools have not been validated but we intended them to better evaluate the cultural idiosyncrasies of South Asian research by using these adapted tools (Roche et al., 2020). These were modified by ST and reviewed by JB and RA. Additionally, RS, a psychiatrist practicing in India, reviewed the items. After modifying the tools, ST and CO independently completed quality appraisals of all the included studies. Any discrepancies were discussed with RA. Studies meeting 50% of the criteria were rated as low quality, 50%–80% as medium quality, and above 80% as high quality.

### Data Synthesis

We conducted a narrative synthesis to report the experiences of adult survivors of CSA, how this impacted their adult lives, and any experiences of treatment or recovery processes. This was conducted by first developing brief textual descriptions for each study and then inspecting the similarities and patterns between the reported findings across the studies. We critically appraised the literature to identify the limitations and merits of the included studies (Popay et al., 2006).

## Results

**Figure 1.**
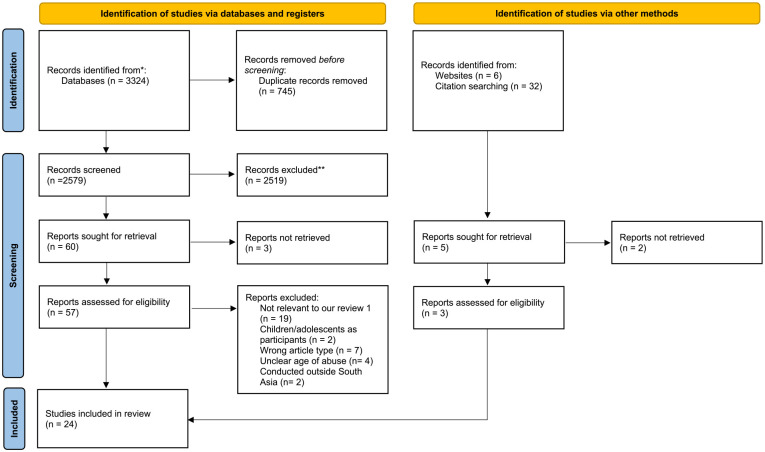
Preferred Reporting Items for Systematic Reviews and Meta-Analyses flow diagram.

We retrieved 3,362 records from databases and journals. After deduplication, we screened 2,541 titles and abstracts to include 65 records for full-text screening. Out of these, we excluded 41 records. The reasons for exclusion were irrelevance to our review, children/adolescents as participants, wrong article type, unclear age of abuse, or conducted outside of South Asia. We included 24 studies in the final review and synthesis. For further details, refer to the PRISMA diagram (Figure 1).

None of the search results were in a language other than English. Hence, the language limit did not impact the inclusion of records and any loss of cultural nuances in reporting findings. Six of the 24 included studies were of high quality where the methods were appropriate and sensitive to disclosing abuse. Sixteen studies were of medium quality due to issues in the recruitment of participants and design, and less sensitivity to disclosure of abuse. Two studies were of low quality due to lack of sensitivity to disclosure of abuse, not using culturally validated tools, lack of generalizability of results and unclear methods. We decided to include all the studies irrespective of their quality ratings. For detailed quality ratings, refer to Supplemental Appendices A–E.

### Study Characteristics

Of the 24 studies included in our review, 20 studies answered the first review question on the experiences of survivors. Six studies responded to the second review question on treatments and recovery. Overall, two studies addressed both the questions. Among the eight South Asian countries, only India (*n* = 18), Pakistan (*n* = 2), Sri Lanka (*n* = 2), and Nepal (*n* = 2) published research relevant to our review questions. Twenty-three studies were published between 2009 and 2022 and only one in 1992. Further details of the study characteristics and key findings are listed in Table 2. All the studies had corresponding authors with origins and experience of training and/or working in the countries under study. None of the included studies involved adult survivors of CSA in planning or conducting the studies.

**Table 2.** Study Characteristics.

Authors	Country	Study Design	Sample Characteristics			Types of Abuse	Main Findings
			*N*	Age Range/Mean Age	Gender/Sex		
Alafia and Manjula (2020)	India	Case-control	Total: 68 Patient: 34 Control:34	18–31 years	Case: Females: 70.58% Males: 29.42% Control: Females: 70.58% Males: 29.42%	General trauma, physical punishment, emotional and sexual abuse	I: No significant difficulty in emotion regulation in patients with BPD with history of CSA
Jain et al. (1992)	India	Case series	5	17–45 years	Females: 100%	Sexual	I: Anxiety, suicidal ideation, fantasies of violent sexual behavior, concentration difficulties, impulsivity and avoidance, sexual difficulties, unsupportive responses to disclosure
Menon et al. (2016)	India	Cross-sectional	36	18–65 years	Male (13.9%) Female (86.11%)	Sexual	I: Dissociation, suicidal or self-harm behavior, paranoid ideation, identity disturbances, no problems in interpersonal relationships
Rathinam, Singh, Chopra et al. (2021)	India	Cross-sectional	399	Mean age: 20.2 years	Male (43.1%) Female (56.9%)	Sexual	I: Anxiety, insecurity, depression, suicidal ideation, difficulty in disclosing abuse
Rathinam, Singh, Gupta et al. (2021)	India	Cross-sectional	452	18–21 years	Not reported	Sexual	I: Anxiety, insecurity, depression, suicidal ideation, difficulty in disclosing abuse
Sharma (2022a)	India	Qualitative interview	11	Median age: 39 years	Males: 100%	Sexual	I: Problems in intimate and familial relationships, including parenting
Dar and Hasan (2018)	Pakistan	Cross-sectional	51	18–40 years	Females: 100%	Physical, emotional, sexual abuse, sexual harassment, emotional neglect	I: Severe dissociation linked to history of CSA
Farooq and Yousaf (2016)	Pakistan	Cross-sectional	80	17–40 years	Females: 100%	Physical, emotional, sexual abuse, multiple abuse, neglect	I: Alexithymia, dissociation linked to history of CSA
Fernandes et al. (2021)	India	Cross-sectional	9,010	18–23 years	Not clear for adults	Physical, emotional, sexual abuse, physical and emotional neglect	I: No links between harmful substance use and CSA
Fonseka et al. (2015)	Sri Lanka	Cross-sectional	1,252	18–49 years	Males: 100%	Physical emotional, sexual abuse, neglect	I: Sexual IPV linked to CSA experiences
Jangam et al. (2016)	India	Case-control	Total: 709 Patient:609 Control: 100	18–50 years	Females: 100%	Physical, sexual, emotional abuse	I: Depression and obsessive compulsive symptoms linked to CSA
Khan et al. (2021)	India	Case-control	Total:120 Nonsufferers:60 Nightmare sufferers:60	Above 18 years	Nightmare sufferers: Males: 50% Females: 50% Non-Nightmare sufferers: Males: 50% Females: 50%	Physical, emotional, sexual abuse, physical and emotional neglect	I: Nightmares and consequent sleep difficulties linked to CSA
MS et al. (2022)	India	Case-control	Total sample: 67 Case:35 Control:32	Case: 24–43 years Control: 22–38 years	Males: 100%	Physical, sexual and emotional abuse, physical and emotional neglect, bullying, community violence, collective violence	I: CSA history least reported by violent offenders
Reddy et al. (2020)	India	Case-control	Total: 323 Patient: 251 Control: 72	NR	Females: 100%	Physical, emotional and sexual abuse	I: CSA not a predictive factor of IPV experiences in adulthood
Sawant and Umate (2021)	India	Cross-sectional	71	15–45 years	Female: 77% Male: 23%	Physical and sexual abuse, coercion	I: More traumatic experiences in adulthood than childhood linked to PNES
Sharma (2022b)	India	Qualitative interview	11	26.53 years	Males: 100%	Sexual	I: Problem in intimate and familial relationships, including parenting, mostly unpleasant responses to disclosure, sexual identity issues, barriers to disclosure
Wesley and Manjula (2015)	India	Case-control	Total: 83 Patient: 53 Control:30	18–60 years	NR	Physical, emotional and sexual abuse, general abuse	I: Depressive symptoms, early maladaptive schemas (subjugation)
Rajkumar and Kirkpatrick (2015)	India	Cross-sectional	62	19–54	Male (50%) Female (50%)	Physical, emotional and sexual abuse	I: No psychotic symptoms in people with schizophreni a linked to CSA
Guragain and Ghimire (2017)	Nepal	Case series	4	Above 18 years	Females: 100%	Sexual	I: Anxiety, fear of revictimization, depressive symptoms, suicidal ideation, self-blame, fear of contamination and compulsive handwashing, somatic complaints II: CBT techniques, individual or group treatment focused on better future and protection from perpetrators
Mujawar et al. (2021)	India	Case report	1	23 years	Female only	Sexual	I: Sexual difficulties, problems in maintaining intimate relationships II: CBT techniques for “maintaining self-control and libido,” no specific model suggested; prescribing fluoxetine
Grover et al. (2008)	India	Case report	1	29 years	Female: 100%	Sexual	II: CBT Techniques, trauma focused, improving self-esteem, decision making
Grover & Singh (2005)	India	Case report	1	19 years	Female: 100%	Sexual	II: CBT Techniques, trauma focused, improving self-esteem, emotional difficulties
Chouliara and Narang (2017)	India	Qualitative interview	20	24–54 years	Females: 70% Males: 30%	Sexual	II: Journey of recovery in four core stages: The Affected Self, Accurate Symbolization, Activation of the Recovering Self, and Self Re-connection, Integration and Growth
Hakkim and Deb (2021)	India	Case series	3	18–25 years	Females: 66.67% Males: 33.33%	Physical, emotional, sexual abuse	II: Meaning making of childhood trauma, development of resilience and self-concept; impact on interpersonal relationships could be a key area in psychotherapy

*Note.* BPD = borderline personality disorder; CBT = Cognitive behavioral therapy; CSA = childhood sexual abuse; IPV = intimate partner violence; PNES = Psychogenic non epileptic seizures. Symbols: I = Question 1 that is, impact of CSA; II = Question 2 that is treatments; NR = Not reported..

Among the 15 quantitative studies, there were 9 cross-sectional and 6 case-control studies. Twelve of these quantitative studies reported the prevalence of abuse and/or neglect, ranging between 1.25% and 44.0%. Except for three studies specifically on CSA, others focused on early traumatic experiences and adverse childhood experiences (ACEs). Among the nine qualitative studies, we identified three interview studies, three case reports, and three case series. Of these, all except one focused exclusively on CSA survivors.

There were nine studies with only female participants and four with only male participants. Eleven studies reported CSA experiences among mixed-gender samples. Only three studies cited the sexual orientation of participants. Socioeconomic status reported in four studies ranged between upper middle and lower classes. Only one study provided information on the religion of participants.

A total of seven studies provided information about perpetrators. Extended family members were cited as perpetrators in five studies, friends and neighbors reported in four studies, and immediate family members and strangers reported in two studies each. Four studies included male perpetrators and only one study mentioned female perpetrators.

Limited studies cited a definition of CSA, but they included contact as well as noncontact CSA. Various instruments were used to assess traumatic experiences, including childhood abuse. These instruments were the Traumatic Experiences Checklist, the Child Abuse Interview, the ACEs —International Questionnaire, the Juvenile Victimization Questionnaire, and the Childhood Trauma Questionnaire. Other instruments used were the Composite Abuse Scale, the Early Trauma Inventory—Self Report Short Form, the International Society on Prevention of Child Abuse Screening Tool—Retrospective version, and the Early Trauma Inventory Self-Reported Short Form. In addition, some studies developed and used semistructured questionnaires based on Alper’s et al. (1993) childhood physical and sexual abuse and Halpérin’s et al. (1996) CSA study. Six studies did not administer validated assessment measures of having experienced CSA but included participants with self-reported CSA.

### Synthesis

#### Question 1: Impacts of CSA on Adult Survivors

The 20 studies that addressed the first research question have been synthesized and grouped into four categories (Mental health conditions and substance use, Impact of CSA on interpersonal relationships, Society’s response to the disclosure of CSA, CSA experiences, and offending behavior) as follows:

##### Mental Health Conditions and Substance Use

A total of 15 studies investigated child abuse and neglect experiences in adults diagnosed with mental health conditions and substance use problems. Of these, 11 studies provided evidence for, and four studies offered evidence against CSA and consequent mental health and substance use problems in adult life. We categorized them into (a) emotional difficulties, (b) risk to self, (c) cognitive and behavioral difficulties, (d) physiological difficulties, and (e) substance use problems.

###### Emotional Difficulties

Twelve studies assessed emotional problems in CSA survivors. Alexithymia, specifically difficulty in verbalizing emotions, and dissociation in adult female participants were predicted by CSA experiences in studies in a Pakistani city (Dar & Hasan, 2018; Farooq & Yousaf, 2016) and a South Indian state (Menon et al., 2016). Feelings of anxiety among CSA survivors were a common problem across an Indian University, females at a Nepalese orphanage, and clinically diagnosed women in India (Rathinam, Singh, Chopra et al., 2021; Rathinam, Singh, Gupta et al., 2021; Guragain & Ghimire, 2017; Jain et al., 1992). In some cases, this was linked to feelings of insecurity (Rathinam, Singh, Chopra et al., 2021; Rathinam, Singh, Gupta et al., 2021), threatening calls from perpetrators’ families, and fear of revictimization (Guragain & Ghimire, 2017). Studies in a university and with the clinical population with CSA history found depressive symptoms like low mood, low self-esteem, anhedonia, and irritability (Guragain & Ghimire, 2017; Jangam et al., 2016; Rathinam, Singh, Chopra et al., 2021; Rathinam, Singh, Gupta et al., 2021; Wesley and Manjula, 2015).

Some contradictory findings were also identified in two Indian studies. Traumatic experiences in adulthood rather than in childhood (including CSA) were linked to psychogenic nonepileptic seizures (Sawant & Umate, 2021). No emotion regulation problems were found in patients with borderline personality disorder (BPD) and histories of CSA (Alafia & Manjula, 2020).

###### Risk to Self

Five studies, conducted only in India and Nepal, studied risk to self in the form of suicidal ideation and attempts. Suicidal ideation was one of the main mental health difficulties experienced by CSA survivors at a University in a South Indian state, two clinical patients in an Indian clinic, and some of the female survivors at a Nepalese orphanage (Guragain & Ghimire, 2017; Jain et al., 1992; Rathinam, Singh, Chopra et al., 2021; Rathinam, Singh, Gupta et al., 2021). Recurrent suicidal or self-harm behaviors were noted in Indian CSA survivors with BPD (Menon et al., 2016).

###### Cognitive and Behavioral Difficulties

Six studies cited cognitive and behavioral difficulties, and as above, these were conducted in only India and Nepal. Cognitive difficulties included self-blame and obsessive thoughts of contamination in a Nepalese study (Guragain & Ghimire, 2017), fantasies of violent sexual behavior, difficulty in concentrating in an Indian study with a clinical population (Jain et al., 1992), and early maladaptive schemas, specifically subjugation in clinically depressed survivors in a South Indian state (Wesley and Manjula, 2015). Some of these cognitions were associated with behavioral difficulties, including reckless and impulsive behaviors, avoiding males and public places, and “rude” behavior with elders in an Indian study (Jain et al., 1992) and frequent handwashing in an Indian and a Nepalese study (Guragain & Ghimire, 2017; Jangam et al., 2016). Moreover, there were disturbances in identity and paranoid ideas in CSA survivors diagnosed with BPD in an Indian city (Menon et al., 2016).

No specific psychotic symptoms or any item on the Positive and Negative Syndrome Scale were associated with physical, emotional, or sexual abuse in childhood in patients diagnosed with schizophrenia at a South Indian institute (Rajkumar & Kirkpatrick, 2015).

###### Physiological Difficulties

Four studies reported physiological difficulties. Nightmares, leading to sleep difficulties, were associated with CSA experiences in students at a University in Delhi (Khan et al., 2021). Sexual difficulties in CSA survivors such as the inability to restrain physical intimacy (Jain et al., 1992) and decreased interest in sexual activities (Mujawar et al., 2021) were cited in two Indian studies. Somatic complaints by prolonged CSA survivors in a care home in Nepal included tenderness in the lower abdomen, headaches and weakness (Guragain & Ghimire, 2017).

###### Substance use Problems

Fernandes et al. (2021) did not find any association between harmful substance use in young adults and a history of CSA. Within the reported ACEs, CSA was the least prevalent.

##### Impact of CSA on Interpersonal Relationships

Seven studies discussed the interpersonal dynamics of adult CSA survivors. Difficulty in forming and maintaining intimate relationships by adult male survivors in India (Mujawar et al., 2021; Sharma, 2022b) and difficulties associated with the inability to confide in one sexual partner by an Indian female (Mujawar et al., 2021) were crucial impacts of CSA. Some of these difficulties were further linked to continued engagement with the perpetrators, although rated low in quality in our assessment (Mujawar et al., 2021).

Male CSA survivors in a Sri Lankan study were twice as likely to perpetrate sexual intimate partner violence (Fonseka et al., 2015). Male survivors questioned their sexual identity or orientation in adulthood if abused by same-sex perpetrators (Sharma, 2022a, 2022b).

Impact on family relationships due to experiences of CSA included survivors’ fears being imposed on their children (Sharma, 2022b) and an inability to trust new carers when CSA was perpetrated by previous carers (Guragain & Ghimire, 2017).

Unlike the above findings, the results of two Indian studies did not report any association between relationship difficulties and CSA experiences. One of those studies cited that participants with BPD and a history of CSA experienced stable relationships more often than those without a history of CSA (Menon et al., 2016). The other study reported that women with mood disorders did not have CSA as a predictive factor of their intimate partner violence experiences in adulthood (Reddy et al., 2020).

##### Society’s Response to the Disclosure of CSA

Five studies in our review, all conducted in India, found that the responses of family members and others to survivors’ disclosure of abuse were unsupportive. These responses included silence, dismissal (Sharma, 2022b), poor emotional support from survivors’ families, with family’s efforts to conceal the abuse, forceful marriage with the perpetrator, being blamed for attracting attention, and being labeled as a “loose woman” (Jain et al., 1992). Of note, these two studies (Jain et al., 1992; Sharma, 2022b) were conducted three decades apart but demonstrated similarly unpleasant responses.

Barriers to the disclosure of abuse included fear of negative consequences, feelings of guilt, fear one will not be believed by their family, loyalty to the perpetrator (Rathinam, Singh, Chopra et al., 2021; Rathinam, Singh, Gupta et al., 2021), the expectation to maintain their secret pact with the perpetrator(s), believing that it was something they enjoyed, feeling ashamed of the sexual nature of the event(s), protecting the perpetrator’s reputation, and protecting others from getting upset (Sharma, 2022a).

##### CSA Experiences and Offending Behavior

An Indian study, assessed by us as low on quality of study design, reported more ACEs among offenders as compared to nonoffenders. However, the frequency of CSA was the least prevalent ACE in violent offenders (MS et al., 2022).

#### Question 2: Treatments Offered to the Survivors

Six studies pertaining to treatments and recovery were identified. There were no studies on the acceptability or efficacy of treatments. Findings from the six included studies have been grouped into “psychological treatments,” “pharmacological treatments,” and “recovery and healing.”

Four studies have cited psychological and pharmacological treatments offered to survivors in South Asia (Grover & Singh, 2005; Grover et al., 2008; Guragain & Ghimire, 2017; Mujawar et al., 2021). Notably, these studies have been reported only in India and Nepal. Of these, three studies were case reports (Grover & Singh, 2005; Grover et al., 2008; Mujawar et al., 2021) and one was a case study (Guragain & Ghimire, 2017). There were two further studies that included nonclinical samples, citing phases of recovery, and healing based on survivors’ views.

##### Psychological Treatments

Cognitive behavioral therapy (CBT) techniques were the preferred choice of treatment by clinicians (Grover & Singh, 2005; Grover et al., 2008; Guragain & Ghimire, 2017; Mujawar et al., 2021). In some cases, the treatments were offered in groups (Guragain & Ghimire, 2017) and were trauma-focused (Grover & Singh, 2005; Grover et al., 2008). The goals of psychological treatments involved “maintaining self-control and libido” (Mujawar et al., 2021), envisioning a hopeful future, supporting their religious beliefs, protecting them from perpetrators’ threats (Guragain & Ghimire, 2017), and improving self-esteem, decision-making abilities, and emotional difficulties (Grover, 2005, 2008).

In two of the studies, clinician(s) did not report any existing treatment models or evidence-based practices for formulation and planning treatment (Guragain & Ghimire, 2017; Mujawar et al., 2021). The other two studies cited employing techniques validated in other cultures (Grover & Singh, 2005; Grover et al., 2008). However, these two studies did not recommend the need for culturally adapting and individualizing the treatment to meet the needs and preferences of the client.

##### Pharmacological Treatment

An Indian female who had a history of CSA and was described as exhibiting sexually disinhibited behavior was prescribed fluoxetine (Mujawar et al., 2021). However, this study is on a single individual, and the study design was rated as low in our quality assessment, limiting generalizations from this study.

##### Recovery and Healing

Two qualitative interview studies explored survivors’ recovery processes. A framework of recovery for CSA survivors, without seeking professional help, was described in a nonclinical sample of 20 participants in India (Chouliara & Narang, 2017). In this study, recovery was described in four phases—turmoil and confusion about sexual abuse, awareness or symbolization of abuse happening to them, activating their recovering self, and, finally, reaching a stage of self-reconnection and reintegration (Chouliara & Narang, 2017). Meaning making of childhood experiences was cited as crucial to CSA survivors’ resilience and self-concept (Hakkim & Deb, 2021). Although these studies did not report on the treatments unlike the four preceding studies mentioned in this section, they highlight that many survivors may not seek professional help but may nevertheless find their ways of processing and dealing with their memories of CSA experiences.

## Discussion

Our systematic review is, to date, the first review to synthesize literature on the impact of CSA on adult survivors from South Asian origin residing in South Asia, the current treatments delivered to them, and their efficacy and acceptability. Our review included 24 studies predominantly from Indian states and the remaining from Pakistan, Nepal, and Sri Lanka. Despite no search limits on the date, only one study was published before 2009. Our included studies had male or female survivors who mostly reported male perpetrators. Key findings were that adult CSA survivors in South Asia could experience emotional, cognitive, and behavioral difficulties, risks to self, relationship problems, and unpleasant responses from others to the disclosure of abuse. While the acceptability and efficacy of treatments have not yet been established with adult CSA survivors in South Asia, the included studies cited mostly psychological treatments. Survivors who did not seek formal help had developed their own ways of dealing with memories of CSA experiences. Only six studies were considered of high quality and the majority of the studies were of medium quality.

Future research with adult CSA survivors in South Asia needs to address the current lack of information on any nonbinary participants and limited data on the sexual orientation of participants.

### Impact of CSA on Adult Survivors

Our review highlights that CSA survivors in South Asia experience mental health and other difficulties. Like our findings, previous studies conducted in the USA and the UK have demonstrated associations between CSA and an increased risk of being diagnosed with depression, anxiety, fear of revictimization, and suicidal tendencies (Infurna et al., 2016; Smolak & Murnen, 2002; Steine et al., 2020; Troya et al., 2021). However, expansion of evidence of associations between self-harm and CSA experiences in South Asia is of vital importance. Notably, studies on severe mental illnesses and their association with CSA experiences are still scant in South Asia.

Our findings are comparable to non-South Asian studies, which also found intrusive and recurring thoughts (McElroy et al., 2016), obsessive-compulsive symptoms, impulsivity, identity problems (Caspi et al., 2008; Estevez et al., 2019), and paranoid ideation (Young et al., 2007). However, in our findings, there was no reporting of experiential avoidance or internal reminders (Batten et al., 2001; Rosenthal et al., 2005),flashbacks or PTSD (Lewis et al., 2016), and eating disorders (Sanci et al., 2008), a significant distinction from studies in the UK, the USA, and Australia. Despite physiological difficulties in our review mirroring non-South Asian literature (Smolak & Murnen, 2002), South Asian research has not yet replicated non-South Asian work on physiological symptoms and physical health problems impacted by CSA (Shevlin et al., 2017).

Limited studies until now on dissociation, emotion dysregulation, and psychotic experiences in CSA survivors have signified the need to explore whether these are universal phenomena or manifesting differently among South Asian CSA survivors. Little research to date has explored or established links between survivors of CSA in South Asia and substance misuse.

Difficulties in maintaining relationships with partners and nonperpetrating family members in South Asian survivors, with one exception (Menon et al., 2016), are alike the relationship difficulties demonstrated by survivors in Western literature (Karatzias et al., 2016). Insufficient research in South Asia on survivors’ intimate relationships and limited or no studies on their other relationships such as with peers, colleagues, and family warrants further exploration in the South Asian context.

In our review, abuse was sometimes incestuous, which could complicate the disclosure with feelings of shame or embarrassment (Gilligan & Akhtar, 2006). Normative of South Asian culture, the extensive focus of survivors could be on protecting the family’s reputation (Sawrikar & Katz, 2017) as well as sacrificing one’s desires and self-esteem to avoid interfamilial conflicts (Shariff, 2009). Despite the often incest abuse reported globally, the survivors in the “West” may more often link feelings of shame and guilt to personal responsibility than to family reputation (Griffin, 2020; Hall & Hall, 2016). Due to the limited evidence on disclosure and the absence of studies on assessing guilt, further studies with South Asian survivors are needed to make generalizations from the data.

A single study in South Asia, rated low by us on the quality of study design, is insufficient to deny the evidenced association in non-South Asian countries between CSA experiences and offending behavior in adulthood (Papalia et al., 2018; Topitzes et al., 2011). Future work with offenders in South Asia and their childhood abuse experience could help inform their rehabilitation.

### Treatments and Suggestions

Research on treatments for adult CSA survivors in South Asia was noteworthy for the complete absence of literature in South Asia evaluating the efficacy and acceptability of treatments. Nevertheless, there was evidence, though limited, of current treatments being delivered. Comparing our findings to the validated treatments for survivors in the UK, the USA, Australia, Canada, and New Zealand, our included studies did not cite EMDR as a treatment choice among others. Pharmacological treatment is not recommended for the treatment of PTSD/CPTSD symptoms (NICE Guidelines, 2022), and our included study did not describe the target symptoms of prescribed medicine. Instead of transposing the validated treatments from non-South Asian countries, there is a pressing need to validate treatments for CSA survivors within South Asian countries.

Those studies conducted with nonclinical samples in South Asia demonstrate that survivors could engage in personalized ways of addressing their CSA experiences, outside the purview of a clinic or a charity. Programs to connect with other adult survivors, find mutual support, and assist survivors who are children and adolescents could be a way forward (Attrash-Najjar et al., 2023). Having certified, accessible, country-specific information on CSA available on Government websites could ensure the dissemination of accurate information (NIH National Institute of Aging, 2023). Such websites could include information such as CSA’s impact on adult survivors, sources of support for survivors, and self-guided strategies for mental and physical health support.

Our review establishes the limited documentation of the needs and difficulties of adult CSA survivors and even more limited research on treatments offered to these survivors in South Asia. While a survivor could decide to not seek professional help, those survivors seeking help should be offered validated interventions that are acceptable and efficacious. This could lead to two strategies: (a) developing new interventions from within the South Asian culture and (b) adapting treatments established in “Western” countries to South Asian culture. There is an established evidence base outside of South Asia yet to be considered, adapted and applied in the South Asian population. We propose research into the potential acceptability of these approaches and their adaptation to the needs of South Asian survivors. This could improve the cultural sensitivity of the interventions (Kunorubwe, 2023) offered to South Asian survivors. There is an urgent need to address the increasing needs of vulnerable to abuse adult CSA survivors in the South Asian region.

#### Strengths and Limitations

Our review has several strengths. (a) The research team included clinical academics and academic researchers from diverse ethnic backgrounds. (b) The review includes literature from a diverse sample, under-represented in trauma literature. (c) We developed an extensive search strategy for identifying studies to date from all eight South Asian countries. (d) Quality assessment tools were modified to bridge the gap between tools developed in the West and studies conducted in South Asia. The review also had some limitations which are important to consider. First, despite the modifications to quality assessment tools, they were not standardized. Second, since only a portion of the records were screened by two reviewers at the title and abstract screening stage, it could have led to potential bias in assessing the eligibility of records. We were, however, reassured by hand searching the journals that we did not miss any other studies. Third, our review could have benefited if we had involved survivors in deciding the eligibility criteria. As recommended by NHS England (2022), including survivors in the synthesis could have ensured the reduction of the gap between findings and their actual implementation, in this case in South Asia.

Our included studies also contained limitations. (a) There was a lack of information on survivors’ sexual orientation and an absence of information on participants identifying as nonbinary or transgender, (b) many of these studies (i) did not account for confounders, (ii) had small sample sizes, (iii) reported correlations and not predictions, and (iv) did not consider the preferred language of the target population and/or the sensitive nature of the disclosure of abuse, and (c) the included studies either did not define or had varied definitions of CSA and used a range of assessments. This makes it difficult to meaningfully compare findings between studies. Clarity and agreement on definitions of CSA in South Asia are essential to develop concrete recommendations for research and practice.

#### Implications and Future Directions

Our review confers a moral and ethical responsibility to improve support and services for adult CSA survivors in South Asia, as stated in Table 3.

**Table 3.** Implications of the Review for Practice, Policy, and Research.

Practice	• Enquiring about experiences of sexual abuse in childhood by practitioners must be irrespective of client’s gender. • Recognizing that the current treatments have not yet been validated in South Asian adult CSA survivors will aid practitioners to deliver treatments with caution. • Clients’ experiences of disclosure of abuse need to be assessed by the practitioners while being sensitive in responding to their disclosure during a consultation.
Policy	• Specialized services for adult CSA survivors could improve the increasing burden of mental health conditions in South Asia. • Improving access to sexual and mental health literacy for children and guardians could prevent the incidence of childhood sexual abuse and identification of early signs of abuse victimization.
Research	• At best, Western models are being used in South Asian regions without adequate consideration of their cultural appropriateness. This would imply that the psychological needs of CSA survivors are not being addressed. • Establishing culturally informed evidence base of treatments for adult CSA survivors, potentially cognitive behavioral approaches, is needed.

*Note.* CSA = childhood sexual abuse.

For researchers, treatments based on a cognitive behavioral approach, as reported by our included studies, have the potential to be adapted for use with adult CSA survivors within and across South Asian countries. Assessing the feasibility and evaluating the efficacy and acceptability of the psychotherapy approaches being delivered to adult survivors of CSA in South Asia has emerged as a vital implication. Future studies need to be conducted in the remaining countries (Afghanistan, the Maldives, Bangladesh, and Bhutan) in the South Asian region from which we did not find any published work. Future work needs to employ an emic approach to research with CSA survivors in the South Asian region to understand affective, cognitive, and behavioral manifestations of their early traumatic experiences.

For mental health professionals, asking about the experiences of CSA to all clients, irrespective of gender, is important. These professionals in South Asia need to be wary of a lack of cultural validation of the treatments they are delivering. A major consideration during treatments must be made to the reactions of survivors’ families and friends to the disclosure of abuse. Moreover, during discussions of abuse, reactions of professionals including healthcare and social workers as well as key stakeholders working with CSA survivors in the nonstatutory sector could be equally important.

For policy makers, the recognition of the need for special services/centers for adult CSA survivors could decrease the mental health burden and treatment gaps in South Asia. A preventative strategy could be better access to sexual and mental health literacy at a young age. It would make children more aware of abuse (Shoib et al., 2022) and could facilitate disclosure. Guardians of children could also be trained to identify any signs of abuse in children (Martin & Silverstone, 2016).

Future work should focus on including the specific experiences of nonbinary people and those who are a part of the LGBTQ+ community. Quality assessment tools, developed in the West and more applicable to the studies conducted in those regions, need to be modified and adapted for use in culture-specific studies.

## Conclusion

The mental health impact of CSA on adult survivors of South Asian origin residing in South Asia has been reported in four out of eight countries in the region. Our findings, as stated in table 4, suggest that CSA may or may not lead to the diagnosis of a mental disorder. Nevertheless, evidence suggests that CSA impacts survivors’ intimate and nonintimate relationships, with cultural norms complicating the maintenance of ties with their perpetrators. The survivors experience emotional, cognitive, behavioral, and physiological difficulties, along with risks to self. Finally, our findings reveal that there are currently no evidence-based treatments for survivors in the South Asian region. Our findings offer a valuable synthesis of existing limited research on adult survivors of CSA in South Asia and highlight the pressing need to further capture the views of survivors, key stakeholders, and clinicians practicing in the region, understand survivors’ treatment needs, and culturally tailor trauma-focused treatments for South Asian survivors.

**Table 4.** Critical Findings.

Review Questions	Findings
What is the impact of childhood sexual abuse on the mental health of adult survivors in South Asia?	• CSA often leads to mental health difficulties and problems in relationships in adult life. • Disclosure of abuse in childhood or adult life mostly includes unpleasant responses with negative repercussions for the survivors. • Scant literature has been published on survivors’ experiences of abuse and mental health difficulties.
Which treatment approaches have been offered to adult survivors of childhood sexual abuse in South Asia and what is the efficacy and acceptability of these approaches?	• Current treatments offered are mainly based on the cognitive behavioral approach. • There was no literature on efficacy and acceptability of current treatments offered to survivors in South Asia. • No evidence-based treatments have been developed yet for survivors in South Asia.

*Note.* CSA = childhood sexual abuse.

## Supplemental Material

sj-docx-1-tva-10.1177_15248380241231603 – Supplemental material for What are the Experiences of and Interventions for Adult Survivors of Childhood Sexual Abuse in South Asia? A Systematic Review and Narrative Synthesis Supplemental material, sj-docx-1-tva-10.1177_15248380241231603 for What are the Experiences of and Interventions for Adult Survivors of Childhood Sexual Abuse in South Asia? A Systematic Review and Narrative Synthesis by Shivangi Talwar, Carlos Osorio, Rajesh Sagar, Rebecca Appleton and Jo Billings in Trauma, Violence, & Abuse

sj-docx-2-tva-10.1177_15248380241231603 – Supplemental material for What are the Experiences of and Interventions for Adult Survivors of Childhood Sexual Abuse in South Asia? A Systematic Review and Narrative Synthesis Supplemental material, sj-docx-2-tva-10.1177_15248380241231603 for What are the Experiences of and Interventions for Adult Survivors of Childhood Sexual Abuse in South Asia? A Systematic Review and Narrative Synthesis by Shivangi Talwar, Carlos Osorio, Rajesh Sagar, Rebecca Appleton and Jo Billings in Trauma, Violence, & Abuse
