# Supplementary material for: What are the Experiences of and Interventions for Adult Survivors of Childhood Sexual Abuse in South Asia? A Systematic Review and Narrative Synthesis
Source: Trauma Violence Abuse. 2024 Feb 22;25(4):2957–71. doi: 10.1177/15248380241231603 (PMC11370205; doi:10.1177/15248380241231603)
Supplement: sj-docx-1-tva-10.1177_15248380241231603 – Supplemental material for What are the Experiences of and Interventions for Adult Survivors of Childhood Sexual Abuse in South Asia? A Systematic Review and Narrative Synthesis [file sj-docx-1-tva-10.1177_15248380241231603.docx]

**Appendices**

**Appendix A** Quality Assessment of Interview Studies using CASP

| **Author(s), Year** | **1** | **2** | **3** | **4** | **5** | **6** | **7** | **8** | **9** | **10** | **11** | **Quality Ratings** |
| --- | --- | --- | --- | --- | --- | --- | --- | --- | --- | --- | --- | --- |
| **Chouliara & Narang, 2017** | + | + | + | / | + | / | + | / | + | + | + | High |
| **Sharma, 2022b** | + | + | + | + | + | - | + | / | + | + | + | High |
| **Sharma, 2022a** | + | + | + | + | + | - | + | / | + | + | + | High |

*Note* Criteria: 1: Was there a clear statement of the aims of the research?, 2: Is a qualitative methodology appropriate?, 3: Was the research design appropriate to address the aims of the research?, 4: Was the recruitment strategy appropriate to the aims of the research?, 5: Was the data collected in a way that addressed the research issue?, 6: Has the relationship between researcher and participants been adequately considered?, 7: Have ethical issues been taken into consideration?, 8: Was the data analysis sufficiently rigorous?, 9: Is there a clear statement of findings?, 10: How valuable is the research?, 11: Were the methods sensitive to the disclosure of history of abuse? Were any special steps taken to ensure the confidentiality of data and/or referral for ensuring any help? Symbols: + = Yes, - = No, / = Partially yes.

**Appendix B** Quality Assessment of Cross-Sectional Studies using AXIS

| **Author(s), Year** | **1** | **2** | **3** | **4** | **5** | **6** | **7** | **8** | **9** | **10** | **11** | **12** | **13** | **14** | **15** | **16** | **17** | **18** | **19** | **20** | Quality Ratings |
| --- | --- | --- | --- | --- | --- | --- | --- | --- | --- | --- | --- | --- | --- | --- | --- | --- | --- | --- | --- | --- | --- |
| Dar & Hassan, 2016 | + | + | - | + | + | + | - | + | + | + | + | + | - | - | + | + | + | + | - | / | Medium |
| Farooq & Yousaf, 2016 | + | + | - | + | + | + | - | + | / | + | + | + | - | - | + | + | + | + | - | + | Medium |
| Fernandes et al. (2021) | + | + | + | + | + | + | - | + | + | + | + | + | - | - | + | + | + | + | - | / | Medium |
| Fonseka et al. (2015) | + | + | + | + | + | + | - | + | + | + | + | + | / | - | + | + | + | + | - | + | High |
| Sawant & Umate, 2021 | + | + | + | + | + | + | / | + | - | - | + | + | ? | - | + | + | + | + | - | ? | Medium |
| Rajkumar & Kirkpatrick, 2015 | + | + | - | + | + | + | - | + | - | + | + | + | - | - | + | + | + | + | - | - | Medium |
| Menon et al, 2016 | + | + | + | + | + | + | - | + | + | ? | - | + | - | - | ? | + | + | + | - | + | Medium |
| Rathinam et al., 2021a | + | + | + | + | + | + | / | + | / | + | ? | + | + | - | + | + | + | + | - | - | Medium |
| Rathinam et al., 2020b | + | + | + | + | + | + | - | + | / | + | + | + | - | - | + | + | + | + | - | / | Medium |

*Note* Criteria 1: Were the aims/objectives of the study clear?, 2: Was the study design appropriate for the stated aim(s)?, 3: Was the sample size justified?, 4: Was the target/reference population clearly defined? (Is it clear who the research was about?), 5: Was the sample frame taken from an appropriate population base so that it closely represented the target/reference population under investigation?, 6: Was the selection process likely to select subjects/participants that were representative of the target/reference population under investigation?, 7: Were measures undertaken to address and categorise non-responders?, 8: Were the risk factor and outcome variables measured appropriate to the aims of the study?, 9: Were the risk factor and outcome variables measured correctly using instruments/measurements that had been trialled, piloted or published previously?, 10: Is it clear what was used to determine statistical significance and/or precision estimates? ( eg. p values, confidence intervals), 11: Were the methods (including statistical methods) sufficiently described to enable them to be repeated?, 12: Were the basic data adequately described?, 13: Does the response rate raise concerns about non-response bias?, 14: If appropriate, was information about non-responders described?, 15: Were the results internally consistent?, 16: Were the results presented for all the analyses described in the methods?, 17: Were the authors' discussions and conclusions justified by the results?, 18: Were the limitations of the study discussed?, 19: Were there any funding sources or conflicts of interest that may affect the authors' interpretation of the results?, 20: Was ethical approval or consent of participants attained? Symbols + = Yes, - = No, / = Partially yes, ? = Can’t tell.

**Appendix C** Quality Assessment of Included Studies using JBI for Case Reports

| **Author(s), Year** | **1** | **2** | **3** | **4** | **5** | **6** | **7** | **8** | **9** | Quality Ratings |
| --- | --- | --- | --- | --- | --- | --- | --- | --- | --- | --- |
| Mujawar et al., 2021 | + | - | - | - | - | N/A | N/A | ? | - | Low |
| Grover & Singh, 2005 | + | + | ? | - | + | + | + | + | + | High |
| Grover et al., 2008 | + | + | + | ? | + | + | + | + | + | High |

*Note* Criteria 1: Were patient’s demographic characteristics clearly described?, 2: Was the patient’s history clearly described and presented as a timeline?, 3: Was the current clinical condition of the patient on presentation clearly described?, 4: Were diagnostic tests or assessment methods and the results clearly described?, 5: Was the intervention(s) or treatment procedure(s) clearly described?, 6: Was the post-intervention clinical condition clearly described?, 7: Were adverse events (harms) or unanticipated events identified and described?, 8:Does the case report provide takeaway lessons?, 9: Were the methods sensitive to the disclosure of history of abuse? Were any special steps taken to ensure the confidentiality of data and/or referral for ensuring any help? Symbols + = Yes, - = No, N/A = Not applicable ? = Unclear.

**Appendix D** Quality Assessment of Included Studies using JBI for Case Series

| **Author(s), Year** | **1** | **2** | **3** | **4** | **5** | **6** | **7** | **8** | **9** | **10** | **11** | **12** | **Quality Ratings** |
| --- | --- | --- | --- | --- | --- | --- | --- | --- | --- | --- | --- | --- | --- |
| Hakkim & Deb, 2021 | + | + | + | - | - | + | - | ? | - | + | N/A | + | Medium |
| Jain et al., 1992 | + | + | + | - | - | + | + | - | ? | + | N/A | ? | Medium |
| Guragain & Ghimire, 2017 | + | ? | ? | - | + | + | ? | + | ? | + | N/A | + | Medium |

*Note* Criteria 1: Were there clear criteria for inclusion in the case series?, 2: Was the condition measured in a standard, reliable way for all participants included in the case series?, 3: Were valid methods used for identification of the condition for all participants included in the case series?, 4: Did the case series have consecutive inclusion of participants?, 5: Did the case series have complete inclusion of participants?, 6: Was there clear reporting of the demographics of the participants in the study?, 7: Was there clear reporting of clinical information of the participants?, 8: Were the outcomes or follow up results of cases clearly reported?, 9:Was there clear reporting of the presenting site(s)/clinic(s) demographic information?, 10: Were the clinicians South Asian speaking in participants' preferred language?, 11: Was statistical analysis appropriate?, 12: Were the methods sensitive to the disclosure of history of abuse? Were any special steps taken to ensure the confidentiality of data and/or referral for ensuring any help? Symbols + = Yes, - = No, N/A = Not applicable ? = Unclear.

**Appendix E** Quality Assessment of Included Studies using CASP for Case-control studies

| **Author(s), Year** | **1** | **2** | **3** | **4** | **5** | **6** | **7** | **8** | **9** | **10** | **11** | **12** | **13** | **14** | **Quality Ratings** |
| --- | --- | --- | --- | --- | --- | --- | --- | --- | --- | --- | --- | --- | --- | --- | --- |
| Khan et al., 2021 | + | + | - | / | / | + | + | - | / | / | / | / | ? | - | Medium |
| M.S. et al., 2022 | + | + | / | / | / | + | ? | - | / | / | / | / | ? | - | Low |
| Wesley & Manjula, 2015 | + | + | / | / | / | + | + | - | / | / | / | - | + | + | Medium |
| Jangam et al., 2016 | + | + | / | / | / | + | + | + | / | / | - | - | + | + | Medium |
| Reddy et al., 2020 | + | + | / | / | / | + | + | + | / | / | - | - | + | + | Medium |
| Alafia & Manjula, 2020 | + | + | / | / | / | + | + | - | / | / | ? | - | + | - | Medium |

*Note* Criteria 1: Did the study address a clearly focused issue?, 2: Did the authors use an appropriate method to answer their question?, 3: Were the cases recruited in an acceptable way?, 4: Were the controls selected in an acceptable way?, 5: Was the exposure accurately measured to minimise bias?, 6: Were the participants and researchers from similar backgrounds or could speak same languages?, 7: (a) Aside from the experimental intervention, were the groups treated equally?, 8: (b) Have the authors taken account of the potential confounding factors in the design and/or in their analysis?, 9: How large was the treatment effect?, 10: How precise was the estimate of the treatment effect?11: Do you believe the results, 12: Can the results be applied to the local population?, 13: Do the results of this study fit with other available evidence?, 14: Were the methods sensitive to the disclosure of history of abuse? Were any special steps taken to ensure the confidentiality of data and/or referral for ensuring any help? Symbols + = Yes, - = No, / = Partially yes, ? = Can’t tell.
